# Mr. Leopold Burroughs

**Published:** 1910-06-25

**Authors:** 


Mr. Leopold Burroughs has died at Eltham Road, Lee, S.E. He took his M.R.C.S. in 1881, and his L.R.C.P. in 1884. A Guy's Hospital man, he became afterwards surgeon to St. John's Hospital, Blackheath, and at one time held the post of obstetrician and house surgeon to Guy's Hospital.

